# A New Coumarin-Acridone Compound as a Fluorescence Probe for Fe^3+^ and Its Application in Living Cells and Zebrafish

**DOI:** 10.3390/molecules26082115

**Published:** 2021-04-07

**Authors:** Jiayong Huang, Zhenshuo Yan, Peiling Qiu, Yufeng Mo, Qizhen Cao, Qiuhong Li, Lini Huo, Lichun Zhao

**Affiliations:** College of Pharmacy, Guangxi University of Chinese Medicine, Nanning 530222, China; hjyshjy@126.com (J.H.); yanzhenshuo8@163.com (Z.Y.); qiupeiling1@outlook.com (P.Q.); moyufeng2@outlook.com (Y.M.); zhen1515901786@outlook.com (Q.C.); Li1093136428@outlook.com (Q.L.)

**Keywords:** Fe^3+^ detection, bioimaging, coumarin-acridone compound

## Abstract

A new coumarin-acridone fluorescent probe S was designed and synthesized, and the structure was confirmed with ^1^H/^13^C NMR spectrometry, single-crystal X-ray diffraction, and high-resolution mass spectrometry. This probe has high sensitivity and selectivity for Fe^3+^ over other testing metal ions at 420 or 436 nm in acetonitrile–MOPS (3-Morpholinopropanesulfonic Acid) buffer solution (20.0 μM, pH = 6.9, 8:2 (*v*/*v*)). Under physiological conditions, the probe displayed satisfying time stability with a detection limit of 1.77 µM. In addition, probe S was successfully used to detect intracellular iron changes through a fluorescence-off mode, and the imaging results of cells and zebrafish confirmed their low cytotoxicity and satisfactory cell membrane permeability, as well as their potential biological applications.

## 1. Introduction

Iron, as we all know, plays an indispensable role in biological functions and systems [1]. More importantly, as one of the most important trace elements in living systems, iron is widely involved in a variety of physiological processes in the human body, including enzymatic catalysis [2], proton transfer [3], cell metabolism [4], nucleic acid synthesis [5], and oxygen transport. However, excessive or insufficient intake of iron can cause many diseases, such as cancer, Parkinson’s disease, and iron deficiency [6,7,8,9,10]. Inadequate iron absorption can affect cerebral blood vessel enlargement and lead to mental retardation in children and newborns [11]. These facts bring out the important impact of developing an effective, fast, environmentally friendly, and economical method for detecting iron ions in the field of biological and medical.

Over recent decades, various analytical methods have been developed for iron ion detection, including flame atomic absorption spectrometry (FAAS) [12], graphite furnace atomic absorption spectrometry (GFAAS) [13], inductively coupled plasma–atomic emission spectrometry (ICP-AES) [14], inductively coupled plasma mass spectrometry (ICP-MS) [15,16], colorimetry [17], and so on. However, these methods are not yet commonly available because of their time-consuming detection, complex operations, and expensive equipment. In contrast, fluorescence probes have been thought to be powerful tools for detection due to their superior properties, such as high selectivity, rapid response, low toxicity, real-time monitoring, and low cost [18,19,20].

**Figure 1:**
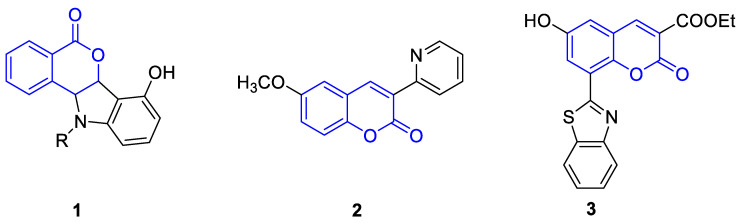
Some coumarin-based fluorescence probes.

The coumarin ring is a well-known fluorophore or chromophore due to its high photostability, large Stokes shift, and intense fluorescence behavior with high quantum yield [21,22,23,24,25,26,27]. A variety of fluorescent probes for detection of Fe^3+^ have been developed based on the coumarin skeleton. In particular, some fragments containing conjugated heterocycles were introduced into coumarin nuclei for new highly sensitive and selective fluorescence probes (Figure 1). For instance, the design and synthesis of a new 4-hydroxyindole-fused isocoumarin (**1**) was described, and it showed fluorescence properties with good quantum yields and fluorescence “turn-off” sensing of Cu^2+^ and Fe^3+^ ions [28]. A new coumarin derivative with an -OCH_3_ and pyridyl substituent (**2**) was proposed as a selective fluorescent sensor for the detection of Fe(III) in CH_3_CN and CH_3_CN:H_2_O (1:1, *v*/*v*) solutions [29]. Peng et al. reported a new coumarin–benzothiazole derivative (**3**), and it served as an ON–OFF-type sensor that displayed a high selectivity and sensitivity for iron ions in a very short time [30].

**Figure 2:**
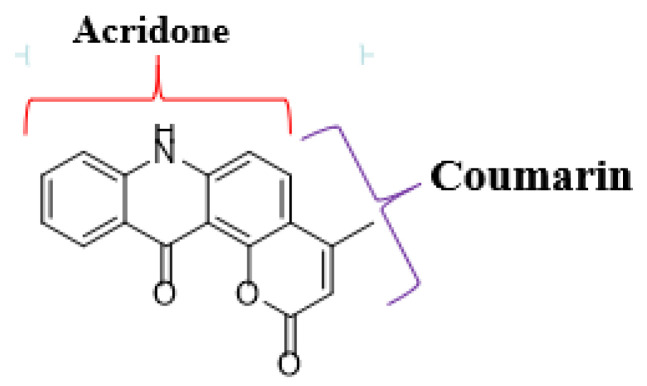
A new structure of coumarin-fused acridone.

In addition, acridones derivatives are mostly used as a dye or fluorescent material owing to their rigid planar structure, excellent optical properties, and photostability [31,32,33]. Based on this, a new fluorescent probe of a coumarin-acridone skeleton (probe S) was designed and synthesized (Figure 2), which contained N and O as the binding sites and larger conjugate coumarin–acridone moieties as the signaling units. Its fluorescent properties and pH titration were systematically studied with fluorescence spectrometry. Additionally, biological testing was then performed as a case to validate this method in biological field detection.

## 2. Materials and Methods

### 2.1. Materials and Instruments

All reagents and metal salts used in the experiment were purchased from Aladdin Industrial Corporation (Shanghai, China) or Sigma-Aldrich Trading Co. LTD (Saint Louis, MO, USA). All of them were reagents used in analytical reagent-grade experiments. Various metal ion stock solutions (Mn^2+^, Mg^2+^, Ag^+^, Na^+^, Ni^2+^, Ca^2+^, Cd^2+^, Co^2+^, Cu^2+^, Fe^3+^, Zn^2+^, Pb^2+^) (0.01 mM) were prepared with the corresponding perchlorate salts. The stock solutions of probe S (20 μM) were prepared in acetonitrile–MOPS (3-Morpholinopropanesulfonic Acid) buffer solution (pH = 6.9, 8:2 (*v*/*v*)). For all measurements, the excitation wavelength was set to 355 nm (slit: 10 nm/10 nm).

^1^H-nuclear magnetic resonance (^1^H-NMR) and ^13^C-nuclear magnetic resonance (^13^C-NMR) spectra were recorded using DMSO-*d*_6_ with a Bruker DRX-400 NMR or Bruker DRX-500 NMR instrument (Rheinstetten, Germany) using tetramethylsilane (TMS) as an internal standard. High-resolution mass spectra (HRMS) were obtained using an Agilent 6210 ESI/TOF mass spectrometer (Palo Alto, CA, USA). The single-crystal X-rays were performed on a Agilent SuperNova, Dual, Cu at zero, AtlasS2 diffractometer (Palo Alto, CA, USA). The melting point of probe S was measured with an X-4 micro-melting point apparatus (Shanghai, China). Fluorescence spectra were recorded on a Shimadzu RF-6000 fluorescence spectrophotometer (Tokyo, Japan) equipped with a quartz cuvette with a path length of 1 cm. The zebrafish were acquired from Shanghai FishBio Co., Ltd. (Shanghai, China). Zebrafish were cultured in a Thermo Scientific Heratherm microbial incubator IMH100-S/IMH100-S SS (Waltham, MA, USA). Confocal laser scanning microscopy (CLSM) imaging was performed on a Leica confocal laser scanning biological microscope SP5 (Wetzlar, German). All pH measurements were made with a PHB-1 pen-type acidity meter (Chengdu, China).

### 2.2. Synthetis of Probe S

**Scheme 1:**
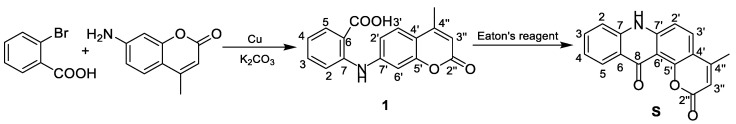
Synthesis of probe S.

The synthesis of the new skeletal coumarin–acridone compound (probe S) is shown in Scheme 1.

#### 2.2.1. The Synthesis of Intermediate **1**

A mixture of 7-amino-4-methylcoumarin (3.50 g, 20 mmol) and 2-bromobenzoic acid (2.01 g, 10 mmol) was added into *N*,*N*-dimethylformamide (50 mL) and stirred until completely dissolving. Then, copper powder (0.64 g, 10 mmol) and potassium carbonate (0.69 g, 5 mmol) were added to the mixture and refluxed at 180 °C for 12 h while being magnetically stirred. After the reaction finished, the reaction solvent was immediately filtered, and the filtrate was acidified to pH 2–3 by aqueous hydrochloric acid solution with a volume ratio of 1:1. A precipitate was produced after staying overnight at 0 °C, and was further recrystallized from chloroform to obtain yellow solid **1** (1.15 g, 77.63% yield), m.p.185.9–186.7 ℃; ESI-MS *m*/*z*: 294.26 ([M − H]^+^), ^1^H NMR (400 MHz, DMSO-*d*_6_) δ 13.28 (s, 1H, -COOH), 9.81 (s, 1H, -NH), 7.96 (d, *J* = 7.9 Hz, 1H, H-2), 7.67 (d, *J* = 8.4 Hz, 1H, H-5), 7.52 (d, *J* = 3.8 Hz, 2H, H-3,3′), 7.18 (d, *J* = 8.3 Hz, 2H, H-4,2′), 7.05–6.91 (m, 1H, H-6′), 6.18 (s, 1H, H-3′′), 2.39 (s, 3H, 4′′-CH_3_); ^13^C NMR (126 MHz, DMSO-*d*_6_) δ 169.84, 160.65, 155.07, 153.74, 145.74, 144.34, 134.54, 132.37, 126.94, 120.67, 117.44, 116.56, 115.60,113.99, 111.40, 104.54, 18.50.

#### 2.2.2. The Synthesis of Probe S

Intermediate **1** (0.60 g, 2 mmol) and Eaton’s reagent (5 mL) were successively added to a 50 mL round-bottom flask. Then, the mixture was heated at 90 °C for 5 h under stirring conditions, whereby the mixture gradually turned into a yellow color. Then, the reaction mixture was poured into 500 mL ice water and was made alkaline with saturated NaHCO_3_ solution, and a precipitate was produced. A yellow solid was prepared by filtering, washing with water, and drying in the air. Then, the crude product was further recrystallized from glacial acetic acid. This gave the product S as a yellow needle crystal (0.44 g, yield: 53.00%), m.p.179.71–82.3 °C; HR-MS (ESI) *m*/*z*: calculated for C_17_H_11_NO_3_ [M + Na]^+^: 300.0631, found: 300.0637; ^1^H NMR (500 MHz, DMSO-*d*_6_) δ 12.01 (s, 1H, -NH), 8.22 (d, *J* = 7.7 Hz, 1H, H-2), 7.99 (d, *J* = 9.0 Hz, 1H, H-3′), 7.76 (dd, *J* = 16.8, 8.6 Hz, 1H, H-3), 7.53 (d, *J* = 8.2 Hz, 1H, H-5), 7.42 (d, *J* = 8.9 Hz, 1H, H-2′), 7.31 (dd, *J* = 14.3, 6.5 Hz, 1H, H-4), 6.34 (s, 1H, H-3′′), 2.46 (s, 3H, 4′′-CH_3_); ^13^C NMR (126 MHz, DMSO-*d*_6_) δ 175.65, 160.33, 154.36, 153.91, 144.86, 139.93, 133.98, 129.90, 126.53, 123.10, 122.64, 117.69, 114.12, 112.44, 111.79, 109.00, 19.18.

#### 2.2.3. Crystal Data and Structure Determination of Probe S 

For further validation of the structure of the target product, a single crystal suitable for an X-ray diffraction study was cultivated from the glacial acetic acid with a slow evaporation method at room temperature. The yellow block crystals of probe S had approximate dimensions of 0.12 × 0.1 × 0.09 mm^3^. All measurements were performed with Cu Kα radiation (λ = 0.71073 Å) on a SuperNova, Dual, Cu at zero, AtlasS2 diffractometer. Out of the 4851 total reflections collected in the range of 4.396° ≤ 2θ ≤ 49.996° (−9 ≤ h ≤ 7, −11 ≤ k ≤ 11, −12 ≤ l ≤ 12) by using w scan mode, 2691 were independent, with R_int_ = 0.0263, of which 1957 were observed with I > 2σ (I) and used in the structure determination and refinements. The structure was solved using direct methods with SHELXS [34] and refined with SHELXL [35]. All non-hydrogen atoms were refined with anisotropic thermal parameters. The hydrogen atoms were placed in the calculated positions. The final full-matrix least-squares refinement gave R = 0.0263, wR = 0.1137 (w = 1/[s^2^(Fo^2^) + (0.0472P)^2^ + 0.1560P], where P = (Fo^2^ + 2Fc^2^)/3, S = 1.007, (Δρ)_max_ = 0.25, and (Δρ)_min_ = −0.27 e/Å^3^.

### 2.3. Fluorescence Analysis

The solutions of the probe S (20.0 μM) in the acetonitrile–MOPS buffer solution (pH = 6.9, 8: 2 (*v*/*v*)) and guest metal cations (0.01 mM) in ultrapure water with a resistivity of 18 MΩ*cm (25 °C) were prepared. Fluorescence spectra were measured by adding various amounts of metal cation solution (10 μL) to the probe S (3 mL) at room temperature. The fluorescence intensity was recorded at λ_ex_/λ_em_ = 355/420 or 355/436 nm, and the excitation and emission slits were both set to 10.0 nm.

### 2.4. In Vitro Cell Imaging

Human cervical carcinoma cells (HeLa) were cultured in DMEM (Dulbecco’s Modified Eagle Medium) containing 10% FBS (fetal bovine serum) and 2% l-glutamine–penicillin–streptomycin, and were kept in a moist atmosphere containing 5% CO_2_ at 37 °C. The cytotoxicity of probe S against HeLa cells was studied using a standard methylthiazolyl tetrazolium (MTT) assay. Then, the cells were transferred to 35 mm confocal dishes in the culture medium for 24 h. For confocal fluorescence imaging, the cells were washed three times with PBS and incubated with probe S (10 μM) for 1 h. One group of cells was treated with medium solution (pH = 7.4, DMSO/DMEM, 1:100, *v*/*v*) for 1 h, while another group was incubated with Fe^3+^ (10.0, 50.0, 100.0 μM) for another 1 h. Cells were washed three times with the PBS buffer solution to remove free compounds before imaging. After that, the cells attached to the confocal culture dish were observed with a confocal scanning system. The excitation wavelength was 355 nm and the fluorescence signals in the range of 400–450 nm were collected.

### 2.5. Fluorescence Imaging in Zebrafish

Typically, 3-day-old zebrafishes were pretreated with probe S (15.0 μM) for 0.5 h in E3 embryo media (34.8 g NaCl, 1.6 g KCl, 5.8 g CaCl_2_·2H_2_O, 9.78 g MgCl_2_·6H_2_O) as a control group. At the same time, the experimental group was incubated with probe S for 0.5 h and then treated with different concentrations of Fe^3+^ (10.0, 50.0, 100.0 μM) for another 0.5 h. After adding 100.0 µL of 1% methylene blue and being cultured for 4 days at 28 °C, it was used for subsequent imaging experiments. The fluorescence images of probe S were collected at 410–460 nm (blue channel).

## 3. Results and Discussion

### 3.1. Synthesis and Structural Characterization of Probe S

**Figure 3:**
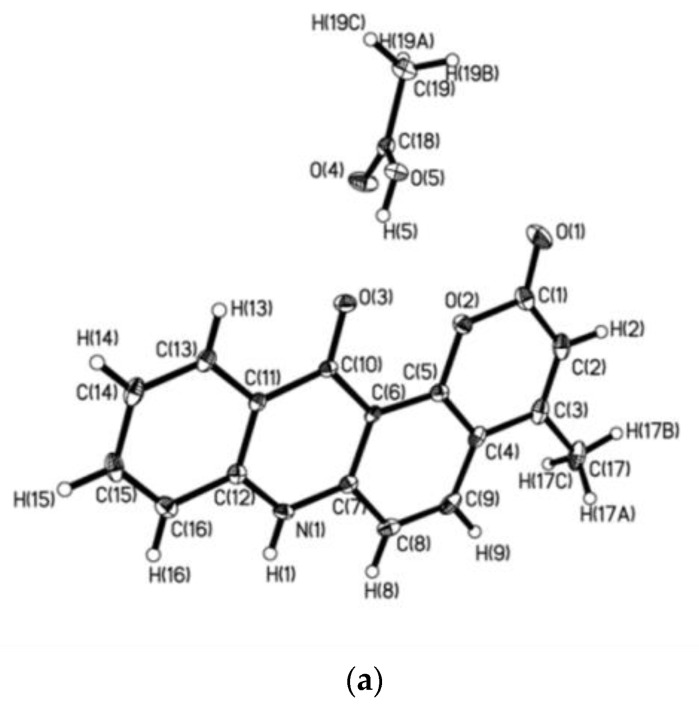

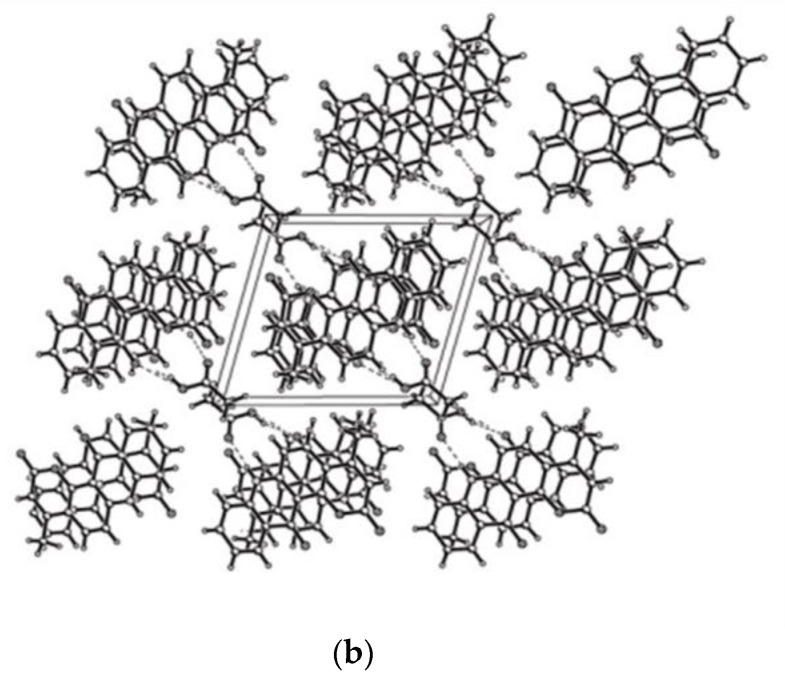
(**a**) X-ray crystal structure and (**b**) the packing of probe S, viewed down from a direction. Dashed lines indicate hydrogen bonds.

The synthetic route to the novel acridone–coumarin fluorescent probe S, as depicted in Scheme 1, was started from Ullmann condensation of 7-amino-4-methylcoumarin and 2-bromobenzoic acid in the presence of a base and copper powder in dimethyl formamide (DMF) to give intermediate **1** (Supplementary Materials). However, it is worth noting that the successful synthesis of intermediate **1** mainly depended on the reaction temperature and ratios of 7-amino-4-methylcoumarin and 2-bromobenzoic acid. The results indicated that the yield was closer to 80% at the reaction temperature of 180 °C and reagent molar ratio of 2:1. Then, the obtained product **1** was cyclized successfully at 90 °C in an Eaton reagent to obtain the corresponding product S in a good yield of 53.00%. However, the crystal of probe S was only purified from glacial acetic acid, and it finally formed an acetate derivative. The structure of the probe S was confirmed by ^1^H NMR, ^13^C NMR, and HRMS. In the ^1^H NMR spectra, the single peak of -NH was observed at δ 12.0, which might be related to the formation of an intermolecular hydrogen bond with acetic acid. The single-crystal X-ray diffraction analysis at 100 (10) K revealed that probe S (CCDC 1983121) crystallized in the triclinic crystal system in space group P-1, and the co-crystal of probe S and the acetic acid molecule was formed in the asymmetric unit (Figure 3). Most C-C bond distances in the main skeleton ranged from 1.347 (3) to 1.466 (3) Å, which fell in the range of single and double bonds. In addition, all bond angles in the plane structure were close to 120°, and these values implied that all four rings of the probe S were in the same plane. In the packing of the crystal structure, the intramolecular hydrogen bond O_5_-H_5_....O_3_ and intermolecular hydrogen bond N_1_-H_1_....O_4_^1^ (^1^1-X,1-Y,1-Z) were formed and were indicated by the distance of 2.604 (2) and 2.784 (2) Å, respectively. That led to a chemical shift of -NH moving to the low field in NMR spectrometry.

### 3.2. Spectral Studies of Probe S

#### 3.2.1. Selectivity Studies for Metal Ions

**Figure 4:**
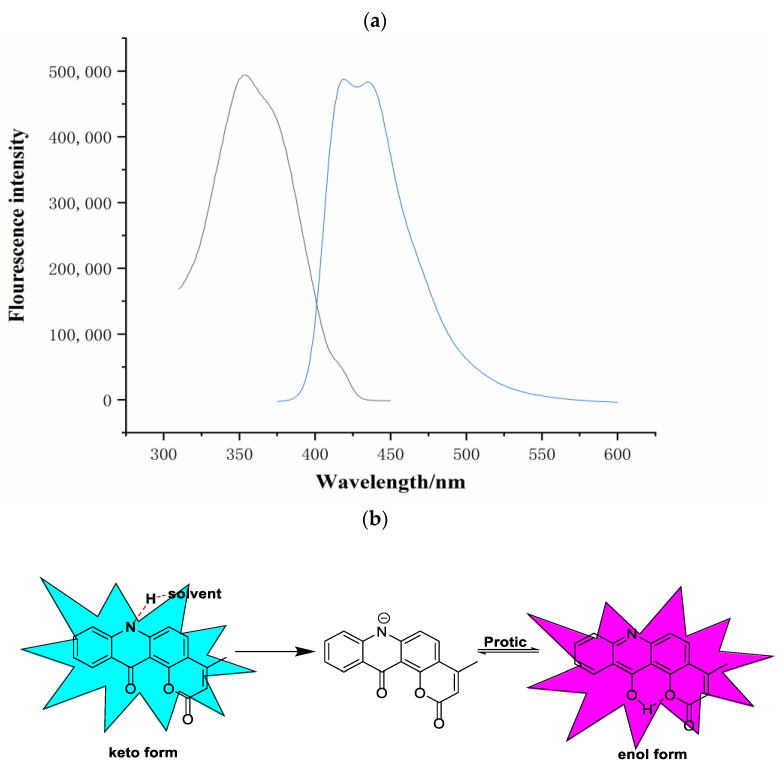
(**a**) Excitation spectra (black line, λ_em_: 420 nm) and emission spectra (blue line, λ_ex_ = 355 nm) of probe S (real values in fluorescence intensity). (**b**) Two proton transfer tautomers of probe S.

The excitation spectra and emission spectra of probe S are shown in Figure 4a. The emission spectra showed dual fluorescence emissions at 420 and 436 nm upon excitation at 355 nm. The emission peaks at the short wave and the long wave corresponded to two proton transfer tautomers (Figure 4b), which was in line with the characteristics of the excited-state intramolecular proton transfer (ESIPT) occurring in most moieties containing intramolecular hydrogen bonds [36]. The process of proton transfer might be as follows: The NH group in the keto form was ionized by forming strong intermolecular hydrogen bonds with some polar solvent (such as CH_3_COOH), and then the proton was transferred to the carbonyl in the same ring, forming an enol form. Similar exchangeable NH protons have been reported in some acridinyl derivatives [37].

**Figure 5:**
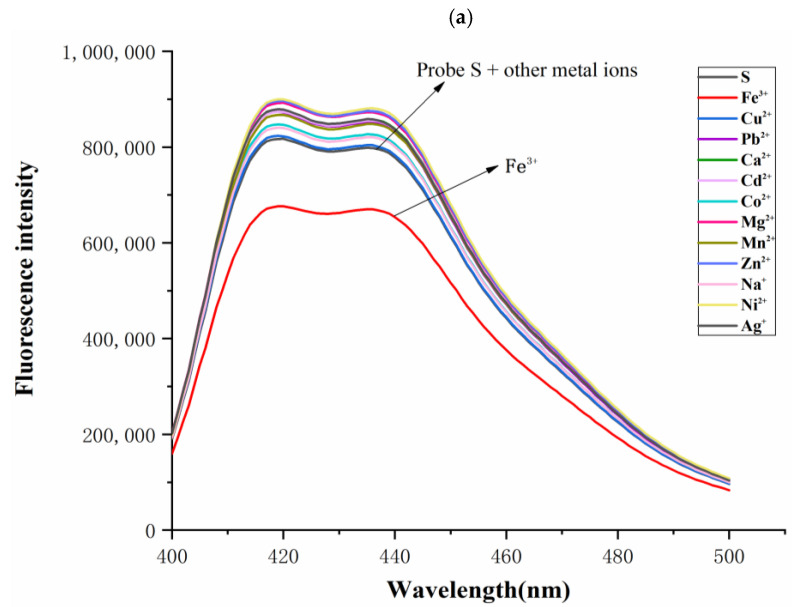

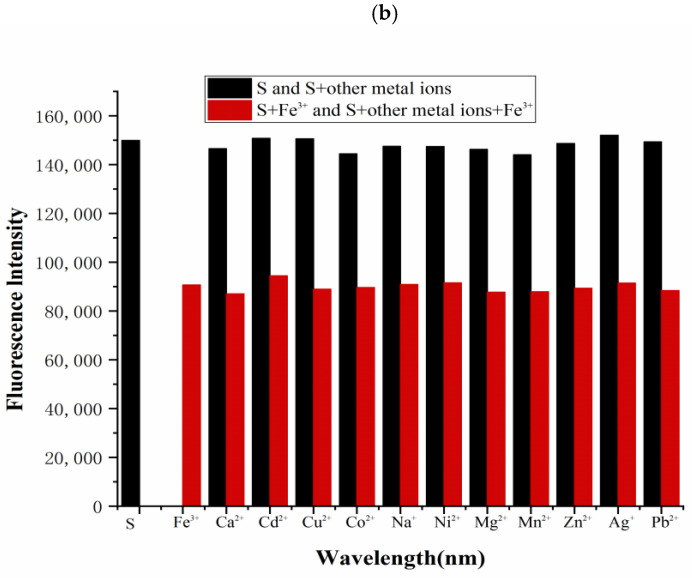
(**a**) The fluorescence responses of probe S (20.0 μM) toward different metal ions (10.0 mM). (**b**) The fluorescent response of probe S (20.0 μM) upon the addition of Fe^3+^ (10.0 mM) in the presence of an excess of various metal ions (10.0 mM) (λ_ex_ = 355 nm, slit: 10 nm/10 nm).

**Figure 6:**
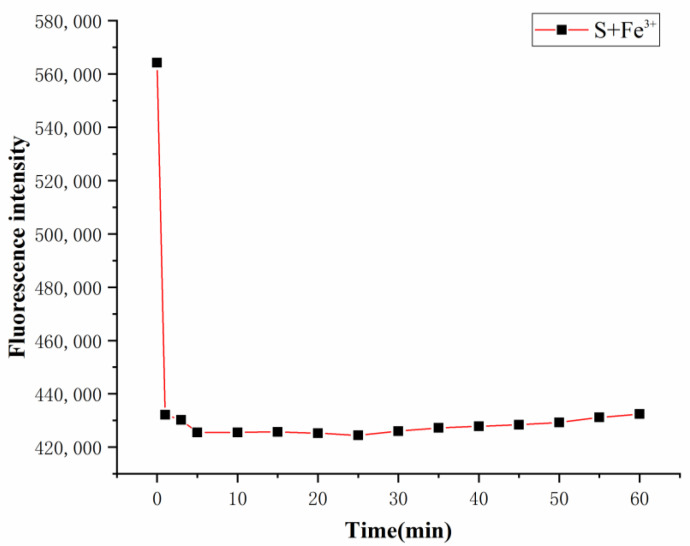
Time course of the fluorescence response of probe S (20.0 μM) in the presence of Fe^3+^ (1 × 10^−2^ M) at room temperature.

The effect of common metal ions on the probe S was investigated with the fluorescence spectra. After adding the metal ion (20 equiv.) to the probe S solutions (3 mL), some metal ions, such as Mn^2+^, Mg^2+^, Ag^+^, Na^+^, Ni^2+^, Ca^2+^, Cd^2+^, Co^2+^, Cu^2+^, Zn^2+^, and Pb^2+^, did not result in any obvious changes in the relative fluorescence intensity. However, there was a notable fluorescence quenching when Fe^3+^ was added, and the fluorescence spectra for different metal ions are shown in Figure 5a. This phenomenon indicated that probe S was more highly selective for Fe^3+^, which is consistent with photo-induced electron transfer (PET) mechanism or π-electron paramagnetic quenching mechanism of Fe^3+^ ions [38,39,40,41]. To further study the selectivity of probe S for Fe^3+^ in the presence of various competing metal ions, the fluorescence spectra of probe S with Fe^3+^ (10.0 equiv.) and the competing metal ions (50.0 equiv.) were examined. As shown in Figure 5b, these coexistent metal cations had almost no significant effects on the fluorescence intensity of probe S for Fe^3+^, which implied its excellent selectivity and strong anti-interference capability. In addition, the response time is a key factor for fluorescent probes, so the time course of the fluorescence response of the probe was recorded (Figure 6). Within the time scale of the test, the fluorescence intensity of probe S (20 μM) changed at 436 nm after the addition of Fe^3+^ (50 equiv.). Fe^3+^ signaling by probe S was very fast, and was actually completed in a few seconds. Subsequently, the fluorescence intensity came to a stable plateau after 5 min, clearly suggesting the good photostability of probe S.

#### 3.2.2. Fluorescence Titration of Probe S with Fe^3+^

**Figure 7:**
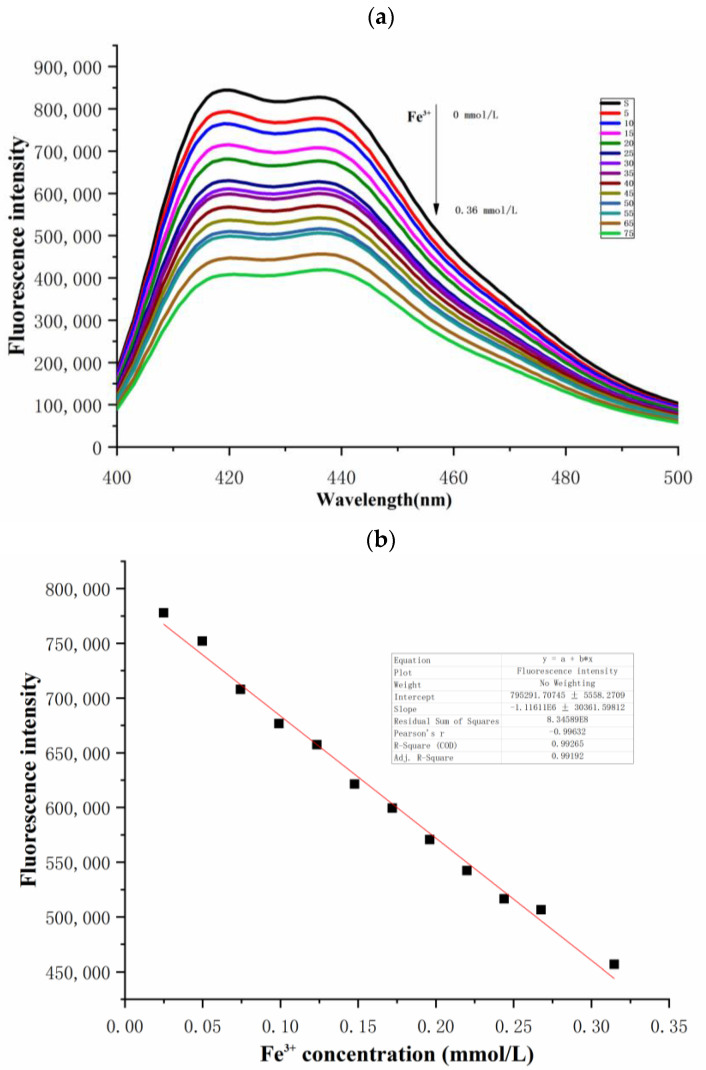
(**a**) Fluorescence response of probe S (20 μM) to different concentrations of Fe^3+^ (0 to 0.35 mM) in acetonitrile–MOPS buffer. (**b**) Linear correlation between the concentrations of Fe^3+^ (0 to 0.35 mM) and corresponding fluorescence intensities (λ_ex_ = 355 nm, λ_em_: 420 nm, slit: 10 nm/10 nm).

A detailed fluorescence titration investigation on the recognition by probe S of different volume of Fe^3+^ (0.0–75.0 equiv.) was carried out. As shown in Figure 7a, the fluorescence intensities of probe S (20.0 μM) at 420 and 436 nm dramatically decreased with small changes in emission upon addition of different concentrations of Fe^3+^, and the detection limit (LOD) was calculated based on that. The fluorescence intensity of probe S without any metal ions was measured at least 10 times to determine the standard deviation of the blank. There existed a good linear relationship between the fluorescence intensity and the final concentration of Fe^3+^ in the range of 0–0.36 mM (R^2^ = 0.99192) (Figure 7b). The detection limit (LOD) was calculated with the equation: LOD = 3σ/K [42], where σ is the standard deviation of blank measurements, and K is the slope between the intensity and sample concentration. The detection limit of probe S was measured to be 1.77 μM according to the equation. This value is much lower than the limits of iron in drinking water and food specified by the US Environmental Protection Agency (EPA) [43].

#### 3.2.3. pH Effect of the Probe for Fe^3+^

**Figure 8:**
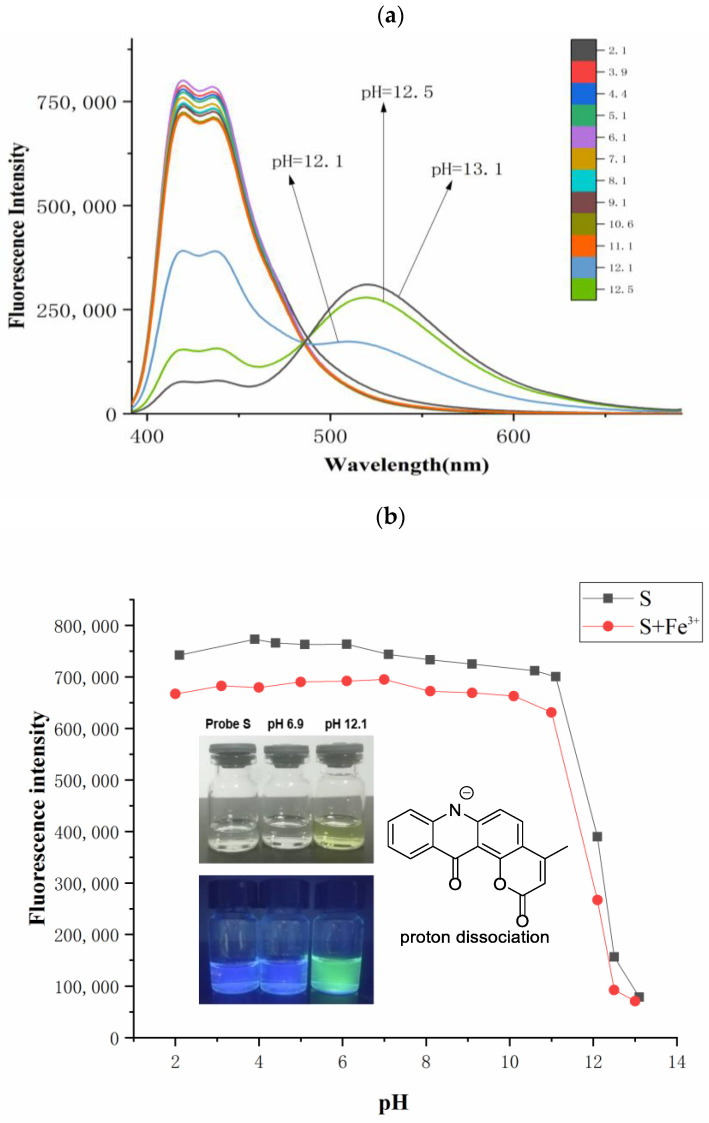
(**a**) The fluorescence response of probe S (20.0 μM) to Fe^3+^ (10.0 mM) at different pH values. (**b**) Fluorescence intensity of probe S (20.0 μM) at various pH values (λ_em_ = 420 nm) in the absence and presence of Fe^3+^ (10.0 mM).

In order to explore probe S as an effective tool for detecting Fe^3+^ at physiological pH, the effect of pH on the fluorescent emission response of probe S to Fe^3+^ was studied by changing the pH value from 2.0 to 13.0 (Figure 8). As seen in Figure 8, the free probe S was very stable within a pH range from 2.0 to 10.1, and there were almost no changes in the emission spectra intensity of probe S in the presence of Fe^3+^ under the same pH values. These results indicate that probe S was very stable under the physiological pH conditions, and it can work in a wide pH range without interference from the pH factors. However, when the pH is equal to 11.0, the fluorescence emissions of two peaks began to decrease. When the pH was adjusted to 12.1, the fluorescence emission peaks of the free probe S and probe S in the presence of Fe^3+^ all displayed a sharp decrease, and a new fluorescence peak appeared, accompanied by a distinct color change from colorless to yellow. The absorption maximum was further shifted to 525 nm with increased intensity when the pH was 13.0, indicating that probe S was very sensitive and formed a new substance in the presence of a strong alkali solution in the range of 12.1–13.0. This might be related to the proton dissociation in the amino group, which forms negative nitrogen ions in the strong alkali solution. It is worth mentioning that the proton dissociation of probe S needs a large amount of alkali because of the formation of intramolecular hydrogen bonds and intermolecular hydrogen bonds with CH_3_COOH in the structure. The obvious increase in the bathochromic shift in strong alkali solution and the molecular structure of probe S proved that excited ESIPT occurred in its molecular skeleton.

### 3.3. Fluorescence Images in Living Cells

**Figure 9:**
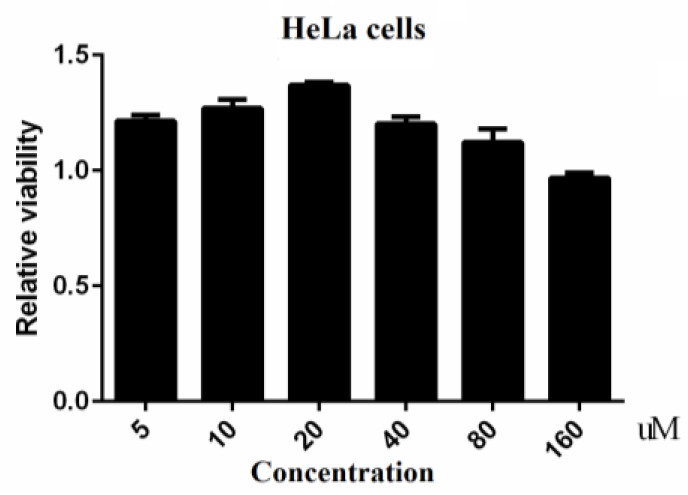
Cell viability of HeLa incubated with varying concentrations of probe S for 48 h.

An MTT assay was used in this study to evaluate the cytotoxicity of probe S against HeLa cells. After incubation of the fluorescent probe in the concentration range of 5–160 μM for 48 h, there were no significant differences in cell proliferation (Figure 9). Therefore, the inhibitory effect of probe S (10 μM) had no influence on the proliferation of cells, and it was then used for further testing of fluorescent imaging in HeLa cells.

**Figure 10:**
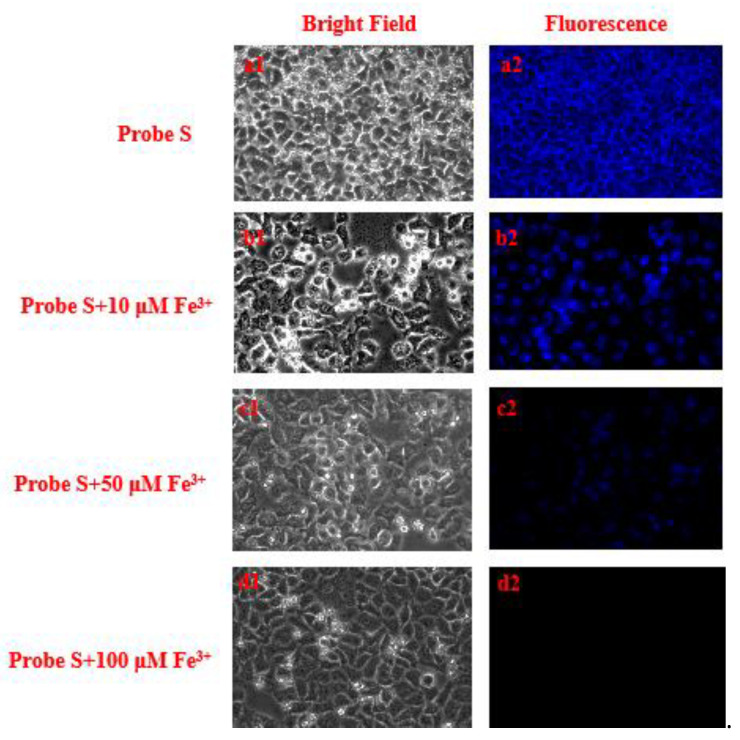
Bright-field and fluorescence images of HeLa cells incubated with probe S (10.0 μM) (**a1**,**a2**) and further incubated with addition of Fe^3+^ (0, 10.0, 50.0, 100.0 μM) (**b2**,**c2**,**d2**); their corresponding bright field images (**b1**,**c1**,**d1**) at 400–480 nm (the scale bar is 20 µm).

**Figure 11:**
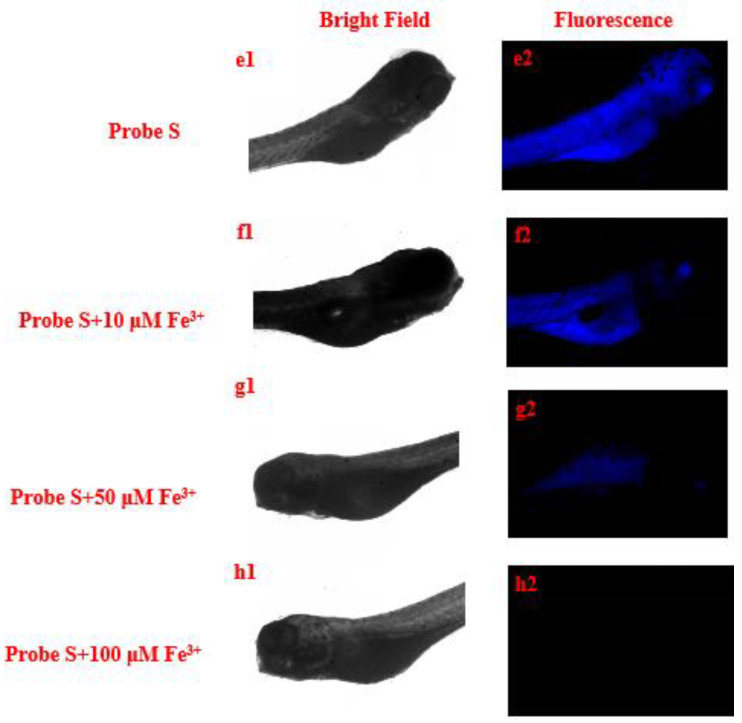
Bright-field and fluorescent images of zebrafish incubated with probe S (10.0 μM) (**e1**,**e2**), further incubated with addition of Fe^3+^ (0, 10.0, 50.0, 100.0 μM) (**f2**,**g2**,**h2**), and their corresponding bright field images (**f1**,**g1**,**h1**) (the scale bar is 500 µm).

To test the capability of probe S of detecting Fe^3+^ in living cells, a confocal fluorescence microscope was used for the fluorescent imaging. As shown in Figure 10(a1–d1), the bright-field images of HeLa cells indicated that the cells were viable in the experimental process. The HeLa cells were first incubated with probe S (10.0 μM) for about 20 min, and a very strong blue fluorescence inside the living HeLa cells was observed (Figure 10(a2)). Subsequently, the cells incubated with probe S were further pretreated with different concentrations of Fe^3+^ (10.0, 50.0, 100.0 μM), and a significant gradient decrease in fluorescence intensity of the blue channel was observed (Figure 11(b2–d2)). After adding 100 μM Fe^3+^, there was no fluorescence from the intracellular area (Figure 11(d2)). The results revealed that probe S could potentially be utilized as a biolabel to respond to Fe^3+^ in HeLa cells.

### 3.4. Fluorescent Imaging in Zebrafish

As a very valuable model organism, zebrafish have been widely used in biological imaging research. Hence, we evaluated the feasibility of probe S for the detection of Fe^3+^ in zebrafish imaging. As revealed in Figure 11, probe S (15 μM) quickly entered the zebrafish body, showing excellent biocompatibility and obtaining a blue fluorescent signal. With the gradual addition of Fe^3+^, the blue fluorescence was significantly reduced, which indicated that probe S could specifically recognize Fe^3+^ in zebrafish. The results implied that probe S has the ability to image Fe^3+^ in zebrafish and further detect Fe^3+^ in living systems.

## 4. Conclusions

In summary, a new coumarin–acridone fluorescence probe S was successfully designed and synthesized via the Ullmann reaction and Friedel–Crafts reaction. Probe S exhibited high selectivity and sensitivity for Fe^3+^, and especially avoided disturbances from common metal ions. Furthermore, it also displayed a good linear relationship between the amount of Fe^3+^ and the fluorescence intensity at 420 or 436 nm, ranging from 0 to 0.35 mM. At the same time, probe S has satisfactory time stability and is very stable under physiological conditions; it can not only penetrate into cells for imaging, but can also penetrate living organs for in vivo imaging. The results show that probe S is a good fluorescence probe and can thus be used to detect Fe^3+^ within living cells and organs.

## Data Availability

Data is contained within the article or Supplementary Materials.

## Supplementary Materials

All the copies of ^1^H NMR, ^13^C NMR, and HR-MS for probe S are available online. CCDC 1983121 contains supplementary crystallographic data for the probe. These datas can be obtained free of charge via http://www.ccdc.cam.ac.uk/conts/retrieving.html (accessed date: 10 February 2020) or the Cambridge Crystallographic Data Centre, 12 Union Road, Cambridge CB2 1EZ, UK; deposit@ccdc.cam.ac.uk; fax: +44-1223-336-033.

## Sample Availability

Sample of the compound S is available from the authors.

## References

[1] Takeda Y., Iwai K. (2016). Maintenance of cellular and body iron homeostasis. Nihon rinsho. Jpn. J. Clin. Med..

[2] Stoyanovsky D.A., Tyurina Y.Y., Shrivastava I., Bahar I., Tyurin V.A., Protchenko O., Jadhav S., Bolevich S.B., Kozlov A.V., Vladimirov Y.A. (2019). Iron catalysis of lipid peroxidation in ferroptosis: Regulated enzymatic or random free radical reaction?. Free Radic. Biol. Med..

[3] Ding C.-W., Luo W., Zhou J.-Y., Ma X.-J., Chen G.-H., Zhou X.-P., Li D. (2019). Hydroxo Iron(III) Sites in a Metal–Organic Framework: Proton-Coupled Electron Transfer and Catalytic Oxidation of Alcohol with Molecular Oxygen. ACS Appl. Mater. Interfaces.

[4] Santiago González D.A.S., Cheli V.T., Wan R., Pabloez P.M. (2019). Iron Metabolism in the Peripheral Nervous System: The Role of DMT1, Ferritin, and Transferrin Receptor in Schwann Cell Maturation and Myelination. J. Neurosci..

[5] Terpilłowska S., Siwicki A.K. (2017). Chromium(III) and iron(III) inhibits replication of DNA and RNA viruses. Biometals.

[6] Bermejo F., Pez S.G.A. (2009). A guide to diagnosis of iron deficiency and iron deficiency anemia in digestive diseases. World J. Gastroenterol..

[7] Wang J., Song N., Jiang H., Wang J., Xie J. (2013). Pro-inflammatory cytokines modulate iron regulatory protein 1 expression and iron transportation through reactive oxygen/nitrogen species production in ventral mesencephalic neurons. Biochim. Biophys. Acta (BBA)-Mol. Basis Dis..

[8] Théevenod F. (2018). Iron and Its Role in Cancer Defense: A Double-Edged Sword. Met. Lons. Life Sci..

[9] Li S.J., Ren Y.D., Li J., Cao B., Ma C., Qin S.S., Li X.R. (2019). The role of iron in Parkinson’’s disease monkeys assessed by susceptibility weighted imaging and inductively coupled plasma mass spectrometry. Life Sci..

[10] Elstrott B., Khan L., Olson S., Raghunathan V., Deloughery T., Shatzel J.J. (2019). The role of iron repletion in adult iron deficiency anemia and other diseases. Eur. J. Haematol..

[11] Torben M., Tina S.R., Lykke T.L. (2018). Iron deficiency and iron treatment in the fetal developing brain-a pilot study introducing an experimental rat model. Reprod. Health.

[12] Machado I., Bergmann G., Pistón M. (2016). A simple and fast ultrasound-assisted extraction procedure for Fe and Zn determination in milk-based infant formulas using flame atomic absorption spectrometry (FAAS). Food Chem..

[13] Aleixo P.C., Nóbrega J.A. (2003). Direct determination of iron and selenium in bovine milk by graphite furnace atomic absorption spectrometry. Food Chem..

[14] Xu T.T., Tian P., Liu S. (2014). Determination of Iron Element in Comb Mushroom by ICP-AES. Appl. Mech. Mater..

[15] Wheal M.S., DeCourcy-Ireland E., Bogard J.R., Thilsted S.H., Stangoulis J.C.R. (2016). Measurement of haem and total iron in fish, shrimp and prawn using ICP-MS: Implications for dietary iron intake calculations. Food Chem..

[16] Abduljabbar T.N., Sharp B.L., Reid H.J., Barzegar-Befroeid N., Peto T., Lengyel I. (2019). Determination of Zn, Cu and Fe in human patients’ serum using micro-sampling ICP-MS and sample dilution. Talanta.

[17] Joao H., Santos N., Icaro A.S., Porto M., Schneider P. (2018). Speciation analysis based on digital image colorimetry: Iron (II/III) in white wine. Talanta.

[18] Lemos V.A., de Carvalho A.L. (2010). Determination of cadmium and lead in human biological samples by spectrometric techniques: A review. Environ. Monit. Assess..

[19] Wang S., Dong X., Dai B., Pan M., He S., Wang J. (2015). Determination of V, Cr, Cu, As, and Pb Ions in Water and Biological Samples by Combining ICP-MS with Online Preconcentration Using Impregnated Resin. J. AOAC Int..

[20] Hao Z.-H., Yao J.-Z., Tang R.-L., Zhang X.-M., Li W.-G., Zhang Q. (2015). Study on the method for the determination of trace boron, molybdenum, silver, tin and lead in geochemical samples by direct current arc full spectrum direct reading atomic emission spectroscopy (DC-Arc-AES). Spectrosc. Spectr. Anal..

[21] Xu Y., Xiao L., Sun S., Pei Z., Pei Y., Pang Y. (2014). Switchable and selective detection of Zn^2+^ or Cd^2+^ in living cells based on 3′-*O*-substituted arrangement of benzoxazole-derived fluorescent probes. Chem. Commun..

[22] Tang Y., Huang Y., Chen Y., Lu L., Wang C., Sun T., Wang M., Zhu G., Yang Y., Zhang L. (2019). A coumarin derivative as a “turn-on” fluorescence probe toward Cd2+ in live cells. Spectrochim. Acta A Mol. Biomol. Spectrosc..

[23] Yu S.Y., Wu S.P. (2014). A highly selective turn-on fluorescence chemosensor for Hg(II) and its application in living cell imaging. Sens. Actuators B Chem..

[24] Wani M.A., Singh P.K., Pandey R., Pandey M.D. (2016). Coumarin-pyrene conjugate: Synthesis, structure and Cu-selective fluorescent sensing in mammalian kidney cells. J. Lumin..

[25] Biswas S., Gangopadhyay M., Barman S., Sarkar J., Singh N.D.P. (2016). Simple and efficient coumarin-based colorimetric and fluorescent chemosensor for F^−^ detection: An ON^1^–OFF–ON^2^ fluorescent assay. Sens. Actuators B Chem..

[26] Li L., Yun S., YuanHui Z., Lan M., Xi Z., Carl R., Gang W. (2016). A single chemosensor for multiple analytes: Fluorogenic and ratiometric absorbance detection of Zn^2+^, Mg^2+^ and F^−^, and its cell imaging. Sens. Actuators B Chem..

[27] Yanar U., Babür B., Pekyilmaz D., Yahaya I., Aydiner B., Dede Y., Seferoglu Z. (2016). A fluorescent coumarin-thiophene hybrid as a ratiometric chemosensor for anions: Synthesis, photophysics, anion sensing and orbital interactions. J. Mol. Struct..

[28] Pathak S., Das D., Kundu A., Maity S., Guchhait N., Pramanik A. (2015). Synthesis of 4-hydroxyindole fused isocoumarin derivatives and their fluorescence “Turn-off” sensing of Cu (II) and Fe(III) ions. RSC Adv..

[29] Pivetta T., Masuri S., Cabiddu M.G., Caltagirone C., Pintus A., Massa M., Isaia F., Cadoni E. (2019). A novel ratiometric and TURN-ON fluorescent coumarin-based probe for Fe(III). New J. Chem..

[30] Peng M.J., Shang H., Yang J.M. (2018). The synthesis and property research of coumarin-benzothiazole fluorescent sensor. Appl. Sci. Technol..

[31] Nikolov P., Petkova I., Köhler G., Stojanov S. (1998). Deactivation processes and hydrogen bonding of excited N-substituted acridones. J. Mol. Struct..

[32] Vezzu D.A.K., Deaton J.C., Shayeghi M., Li Y., Huo S. (2009). Acridinone/Amine(carbazole)-Based Bipolar Molecules: Efficient Hosts for Fluorescent and Phosphorescent Emitters. Org. Lett..

[33] Pereira R.C., Pontinha A.D.R., Pineiro M., De Melo J.S.S. (2019). A comprehensive spectral, photophysical and electrochemical study of synthetic water-soluble acridones. A new class of pH and polarity sensitive fluorescent probes. Dye. Pigment..

[34] Sheldrick G.M. (2008). A short history of SHELX. Acta Cryst..

[35] Sheldrick G.M. (2015). Crystal structure refinement with SHELXL. Acta Cryst..

[36] Yi P.G., Yang X.C., Yu X.Y., Liu Z.J., Liu J., Wang Z.X., Li X.F. (2012). Proton Transfer of 2-(2-Amino-3-pyridyl)-benzimidazole Under the Inclusion Interaction with Cucurbit. Chem. J. Chin. Univ..

[37] Jana T., Ján I., Ivan D., Stanislav B., Pavol K., Jana P., Marián S., Karel D.K. (2008). Regioselectivity and Tautomerism of Novel Five-Membered Ring Nitrogen Heterocycles Formed via Cyclocondensation of Acylthiosemicarbazides. Molecules.

[38] Böttcher R., Langhammer H.T., Kücker S., Eisenschmidt C., Ebbinghaus S.G. (2018). On the incorporation of iron into hexagonal barium titanate: I. electron paramagnetic resonance (EPR) study. J. Phys. Condens. Matter.

[39] Ganeshraja A.S., Karthikeyan V., Krishnamoorthy A. (2020). New monomeric mixed-ligand complex of iron(III)-3-chloropyridine: Synthesis, structure, luminescence, electrochemical and magnetic properties. J. Mol. Struct..

[40] Fuwa Y., Wakeshima M., Hinatsu Y. (2010). Crystal structure, magnetic properties, and Mössbauer spectroscopy of new layered iron oxyselenide Nd_2_Fe_2_O_3_Se_2_. J. Phys. Condens. Matter.

[41] Zhu X., Jiang R. (2010). Determination of Iron(III) by Room Temperature Ionic Liquids/Surfactant Sensitized Fluorescence Quenching Method. J. Fluoresc..

[42] Wang Q., Yang L., Wang H., Song J., Ding H., Tang X.-H., Yao H. (2017). A highly selective and sensitive turn-on fluorescent probe for the detection of Al^+3^ and its bioimaging. Luminescence.

[43] Cha K.-W., Park K.-W. (1998). Determination of iron(III) with salicylic acid by the fluorescence quenching method. Talanta.

