# Supplementary material for: A New Coumarin-Acridone Compound as a Fluorescence Probe for Fe3+ and Its Application in Living Cells and Zebrafish
Source: Molecules. 2021 Apr 7;26(8):2115. doi: 10.3390/molecules26082115 (PMC8067698; doi:10.3390/molecules26082115)
Supplement: Supplementary file 1 [file molecules-26-02115-s001.pdf]

molecules

MDPI

The ^{1}H NMR, ^{13}C NMR and MS datas of intermediate 1 and probe S

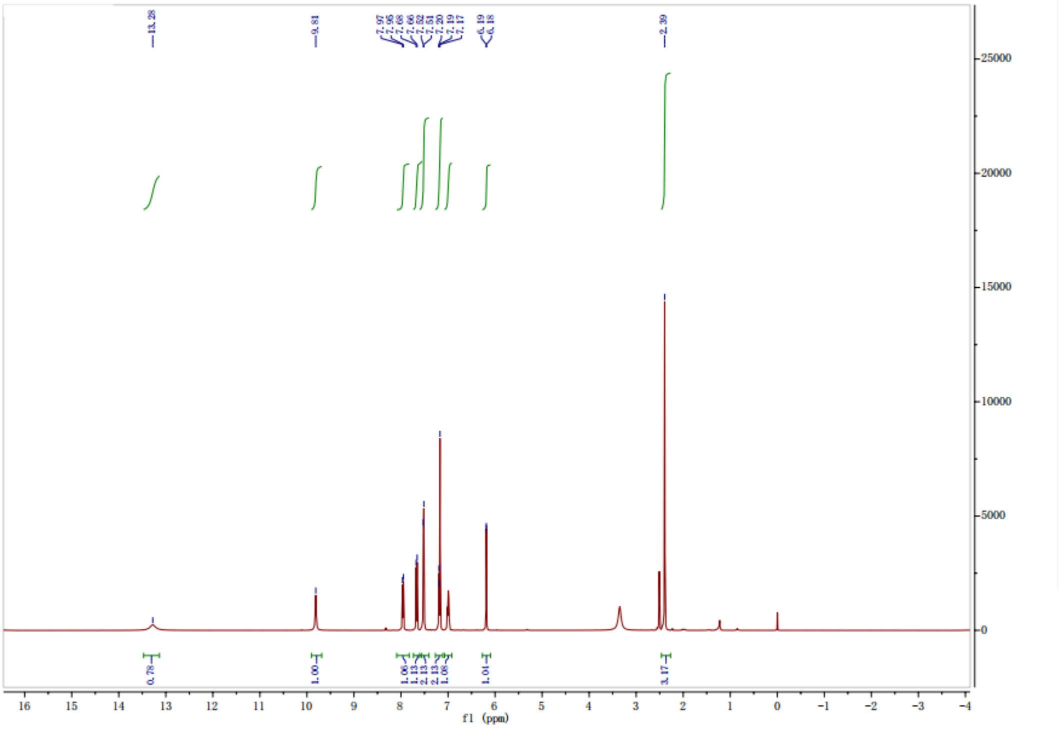

Figure S1 The ^{1}H NMR of intermediate 1

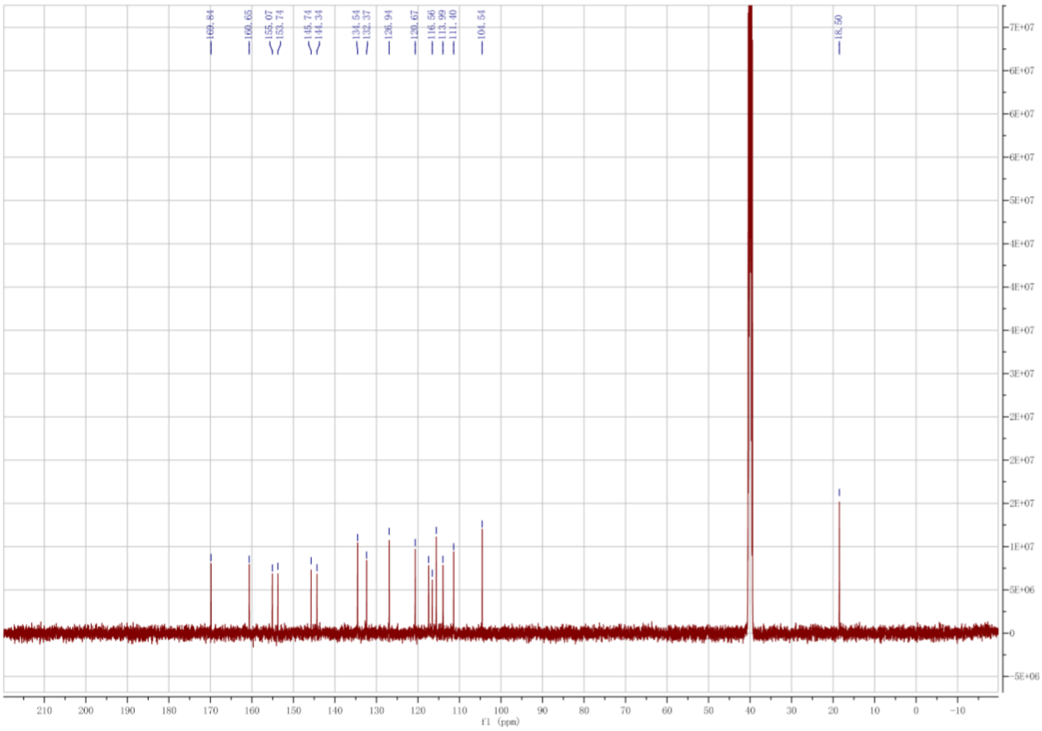

Figure S2 The ^{13}C-NMR of intermediate 1

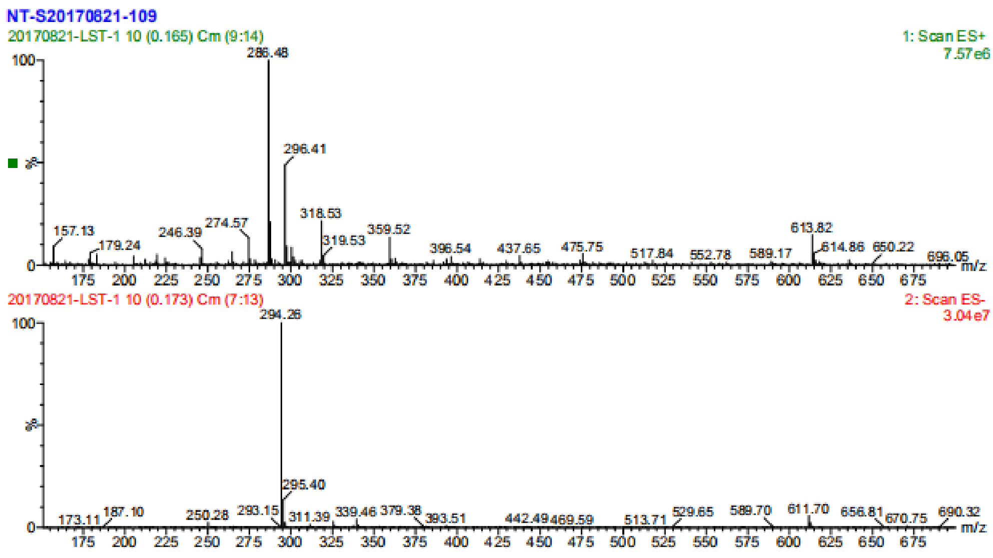

Figure S3 MS Spectrum of intermediate 1

Generic Display Report

Analysis Info Acquisition data:30/08/2019

Analysis name:J-2

Method esi_pos_50-1000_with calibration_for 1min.m operator: WHS

Sample Name: Instrument: Agilent 6210 ESI/TOF MS

Comment

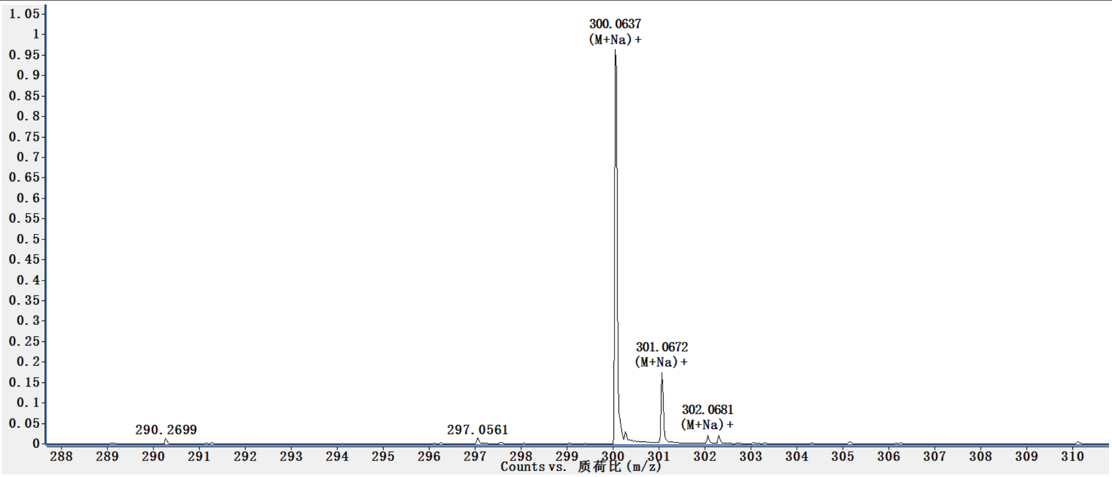

Calculated ms: 300.0631 found: 300.0637

Figure S4 HR-MS Spectrum of probe S

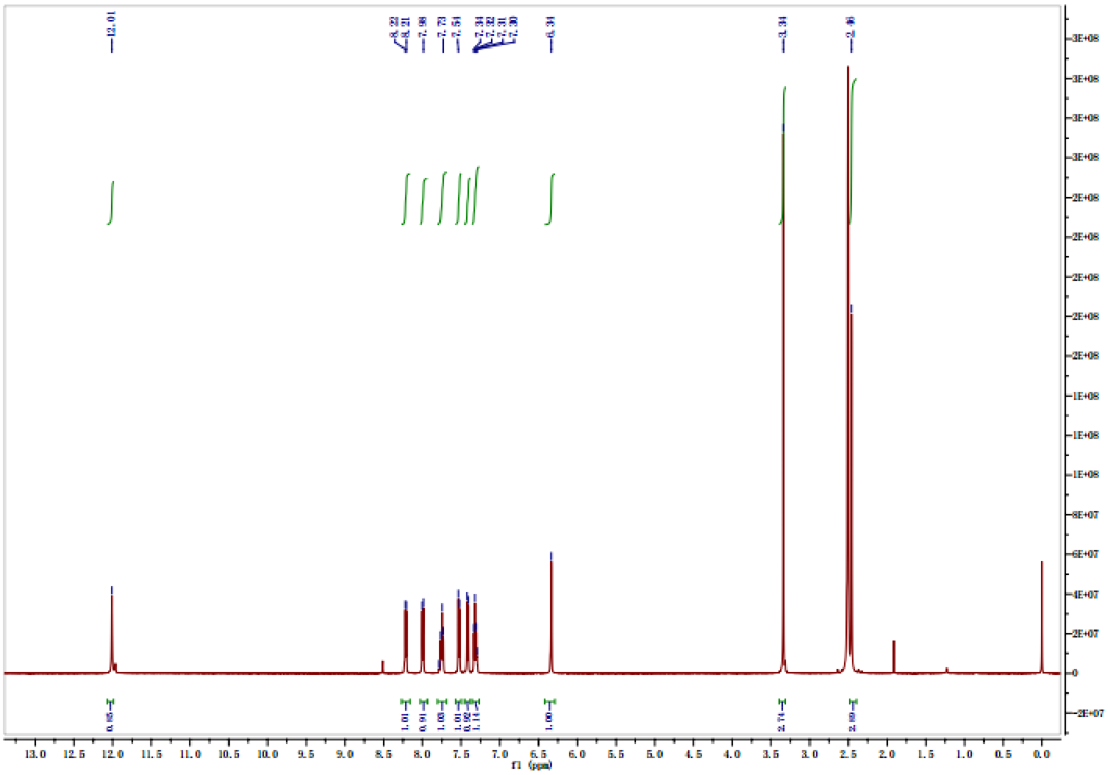

Figure S5 The ^{1}H NMR of probe S

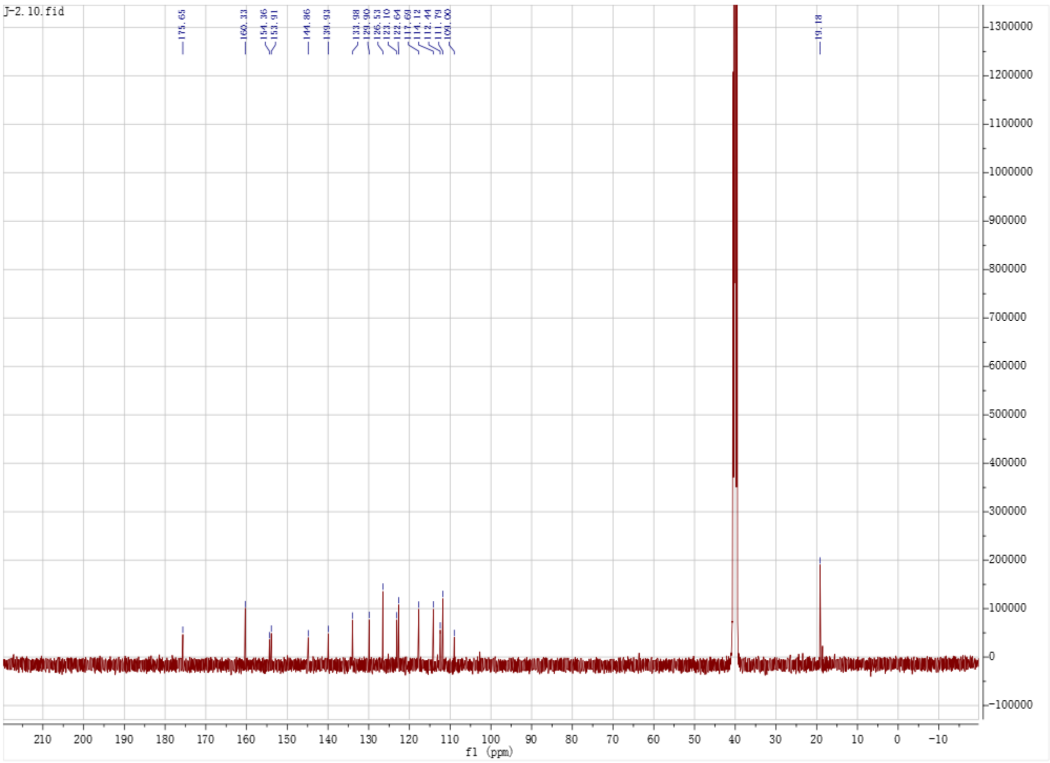

Figure S6 The ^{13}C-NMR of probe S
